# A survey of basal insects (Microcoryphia and Zygentoma) from subterranean environments of Iran, with description of three new species

**DOI:** 10.3897/zookeys.806.27320

**Published:** 2018-12-13

**Authors:** Rafael Molero, Mohadeseh Sadat Tahami, Miquel Gaju, Saber Sadeghi

**Affiliations:** 1 Departamento de Zoología, C-1 Campus de Rabanales, University of Córdoba, 14071 – Córdoba, Spain University of Córdoba Córdoba Spain; 2 Entomology Research Lab, Faculty of Science, Biology Department, Shiraz University, Shiraz, Iran Shiraz University Shiraz Iran

**Keywords:** Archaeognatha, cave fauna, *
Ctenolepisma
*, *
Haslundiella
*, *
Lepidospora
*, Lepismatidae, Machilidae, Nicoletiidae, taxonomy, Thysanura

## Abstract

A survey of wingless insects belonging to the orders Microcoryphia (=Archaeognatha) and Zygentoma (=Thysanura s. str.) has been performed in subterranean habitats of central Iran. As a result, several new species have been discovered. In this work, three new species are described: a new species of bristletail of the family Machilidae, *Haslundiellairanica***sp. n.**, a new silverfish of the family Lepismatidae, *Ctenolepismasubterraneum***sp. n.**, and a new Nicoletiidae, Lepidospora (Brinckina) momtaziana**sp. n.** These new taxa are compared with related species in their respective genera and keys for their identification are provided: one for all known species of *Haslundiella* and one for all basal insects of subterranean environments of Iran which includes those previously reported. Moreover, the previously published keys of Iranian *Ctenolepisma* and the subgenus Brinckina are modified to include the new species. Three additional species of Lepismatidae are reported in this work: *Neoasterolepìsma palmonii* and *Ctenolepismatargionii* are newly recorded from Iran and both species, together with *Acrotelsacollaris*, are cited for the first time in the subterranean habitats. This survey progresses the knowledge on the biodiversity of these insects in Iran.

## Introduction

The subterranean fauna of basal insects is poorly studied in most parts of the world. We consider here as basal insects the orders Microcoryphia (=Archaeognatha) and Zygentoma (=Thysanura s.str.), which both belong to class Insecta. They are primitively wingless and have been included traditionally in the group Apterygota together with Collembola, Protura and Diplura, groups of the superclass Hexapoda that are nowadays excluded from Insecta.

Recently, Iranian caves have been the subject of an extensive faunal study and as a result numerous new invertebrate species, subspecies and even higher taxa are described, most of them known to be highly endemic to only one cave (Christophoryová et al. 2013, Kashani et al. 2013, Malek-Hosseini et al. 2015a, 2015b, 2016a, 2016b, Tahami et al. 2016, 2017a, 2017b, 2017c, 2017d, 2018).

**Figure 1.**
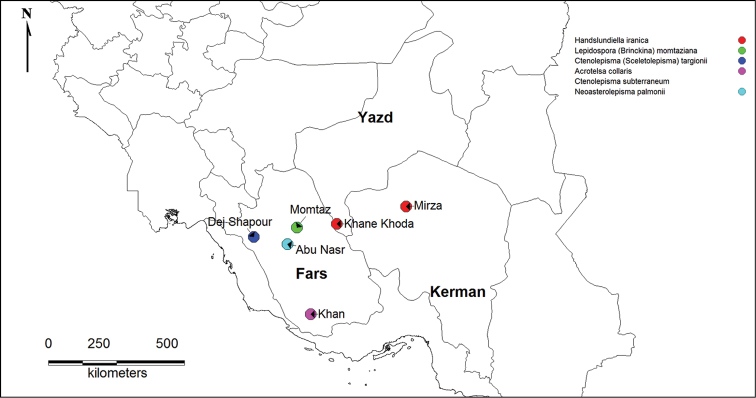
Distribution map of cave localities, Iran.

A basal insects survey has been performed in subterranean environments of the Zagros Mountain ranges and the central zone of Iran, covering a vast area of the center of the country. Fars province (Fig. 1) presented the greatest diversity of species and higher taxa. This is in part due to a higher abundance of caves, favorable climate (such as suitable annual humidity and temperature, sufficient precipitation and soil fertility) and, compared to other provinces, high diversity of all insect groups on the surface.

As a result of this study, eight species belonging to two orders (Microcoryphia and Zygentoma) and three families (Machilidae, Lepismatidae and Nicoletiidae) have been found. Of these eight species, five of them are new; two belong to new supraspecific taxa of the family Nicoletiidae and were described previously (Tahami et al. 2018). The remaining three new species are described in this work – one Machilidae belonging to the genus *Haslundiella* Janetschek, 1954, one Lepismatidae to the genus *Ctenolepisma* Escherich, 1905 and a third species of Nicoletiidae belonging to the genus *Lepidospora* Escherich, 1905 – together with comments on other three species of Lepismatidae that have were found in subterranean habitats, two of them reported for the first time for Iran.

Keys for identifying these eight species of subterranean basal insects in Iran are presented, together with a key to the genus *Haslundiella* (Microcoryphia, Machilidae) and annotations to include the new species of Zygentoma in previously published keys: Kahrarian et al. (2014) for Iranian *Ctenolepisma* and Mendes (2002) for *Lepidospora*.

## Material and methods

### Collecting methods

Specimens were collected during a faunal survey of the caves of Zagros and the Central zone of Iran. Locations where basal insects were collected are shown in Fig. 1. According to the policy of the Iranian Speleological Society, cave coordinates could not be published, for the sake of caves’ safety and conservation. The collection was carried out through a meticulous investigation of all zones of the caves, generally referred to as the endogean (entrance), parahypogean (twilight) and hypogean (dark) zones. Specimens were found mainly in the soil or under stones and collected with a fine brush. After their capture, they were preserved in labeled vials with ethanol 75% and transported to the laboratory for further studies.

### Laboratory material and methods

When dissected, specimens were mounted on a slide using Tendeiro medium (Molero-Baltanás et al. 2000). Identifications were made using a Nikon Labophot light microscope, and for new species, drawings were made using a camera lucida attached to the microscope. Some micrographs were taken using a Nikon DS-Fi1 digital camera on the aforementioned microscope.

Abbreviations for the descriptions of Microcoryphia are explained in the legend of Tables 1 and 2.

The types of the new species are deposited in the collection of the Museo Nacional de Ciencias Naturales in Madrid (MNCN, Spain). The remaining material is deposited in the scientific collection of the Department of Zoology of Córdoba University, Spain (UCO) and in the Zoological Museum and Biological Collection of Shiraz University, Iran (ZM-CBSU).

## Taxonomy

### Order Microcoryphia (=Archaeognatha)

#### Family Machilidae

##### Haslundiella iranica

Taxon classificationAnimaliaMicrocoryphiaMachilidae

Gaju, Molero, Tahami & Sadeghi sp. n.

http://zoobank.org/DF55D262-64C2-47CE-8ED9-0A8834DA25DD

**Figure 2.**
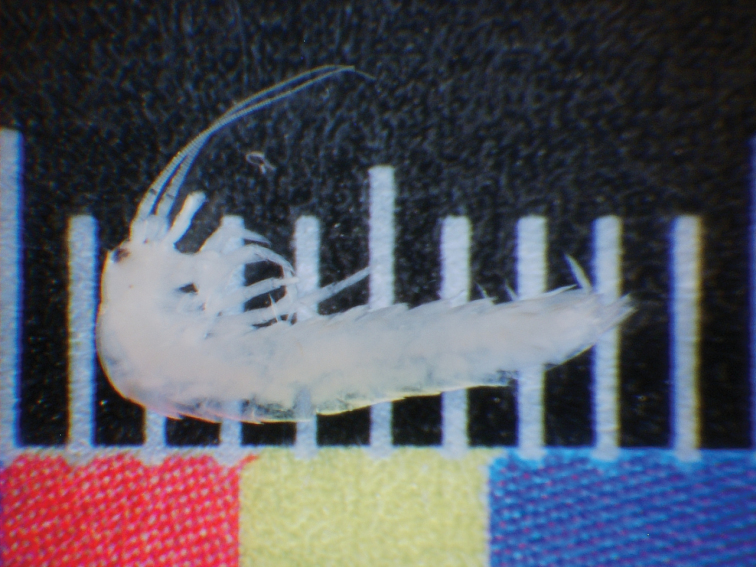
*Haslundiellairanica* sp. n., habitus (female specimen from Mirza cave).

**Figure 3.**
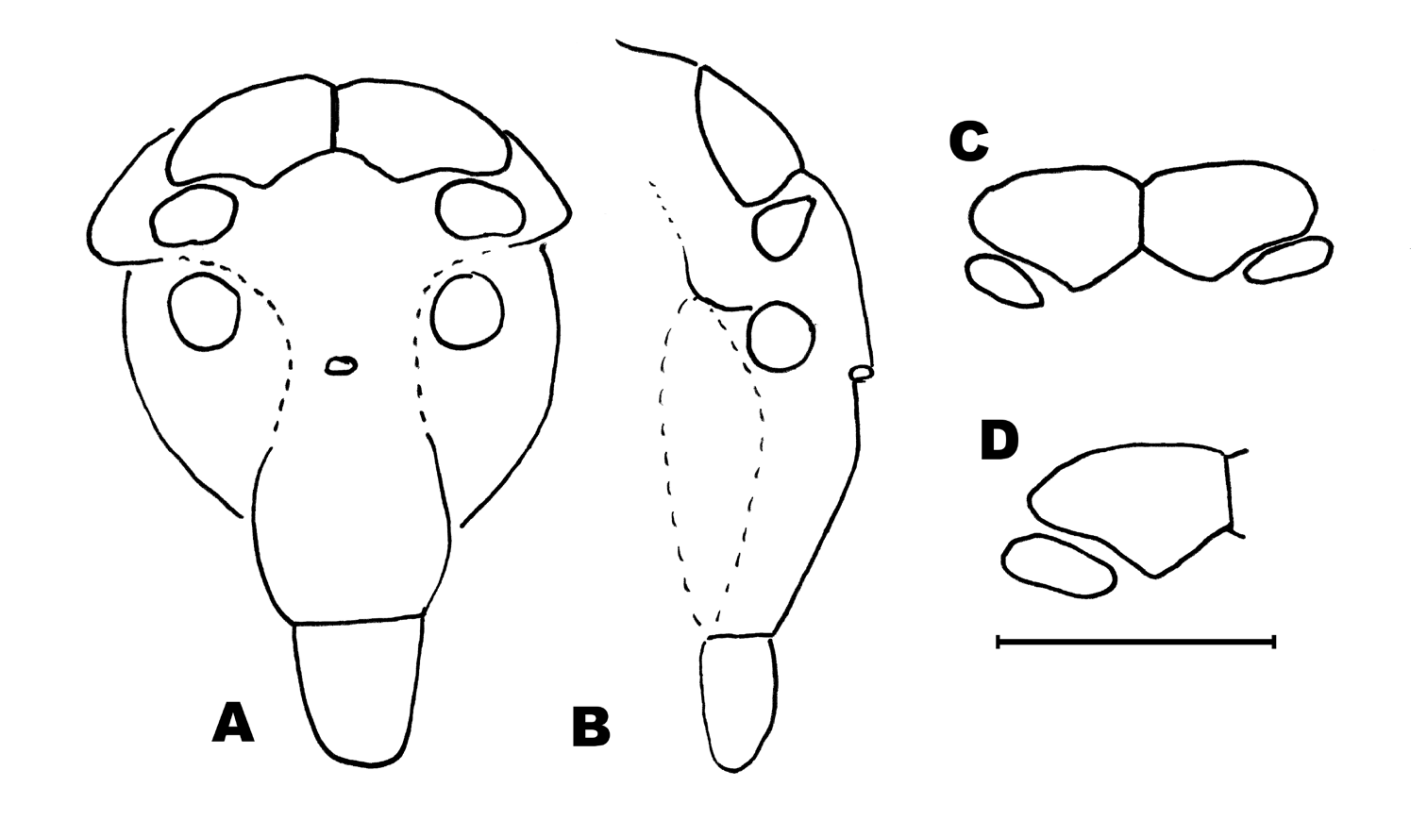
*Haslundiellairanica* sp. n., female from Khane Khoda cave. **A** head frontal view **B** head, lateral view **C** frontal view of compound eyes **D** frontal view of right eye and lateral ocellus. Scale bar: 1 mm.

**Figure 4.**
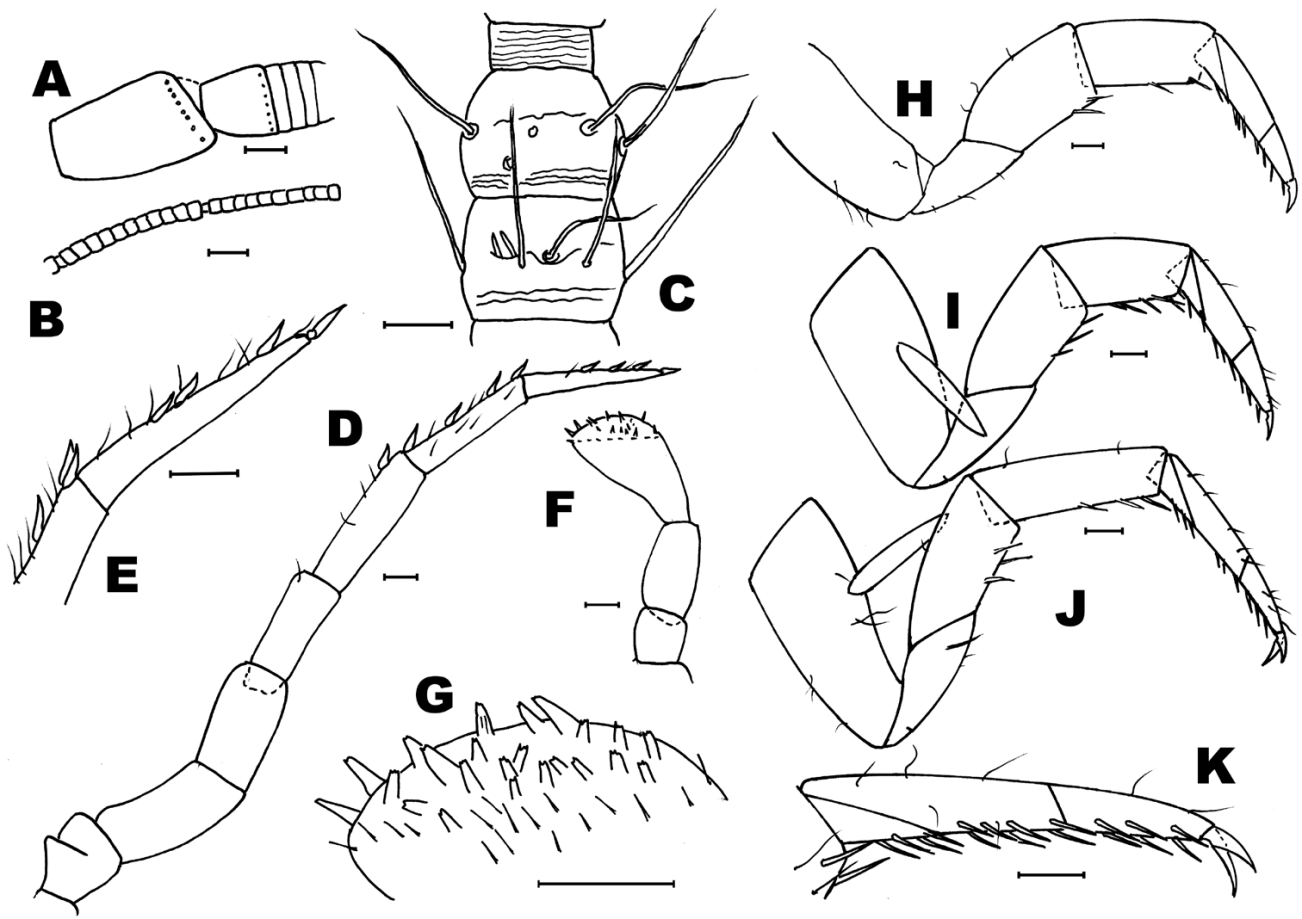
*Haslundiellairanica* sp. n., male holotype (Mirza cave). **A** antennal scapus, pedicellus and basal annuli of flagellum **B** antennal distal chains **C** chaetotaxy of apical annuli of antennal distal chain **D** maxillary palp **E** seventh article of maxillary palp **F** labial palp **G** field of sensorial cones of third article of labial palp **H** fore leg **I** mid leg **J** hind leg **K** tarsus of hind leg. Scale bars: 0.1 mm (**A, B, D–K**); 50 μm (**C**).

**Figure 5.**
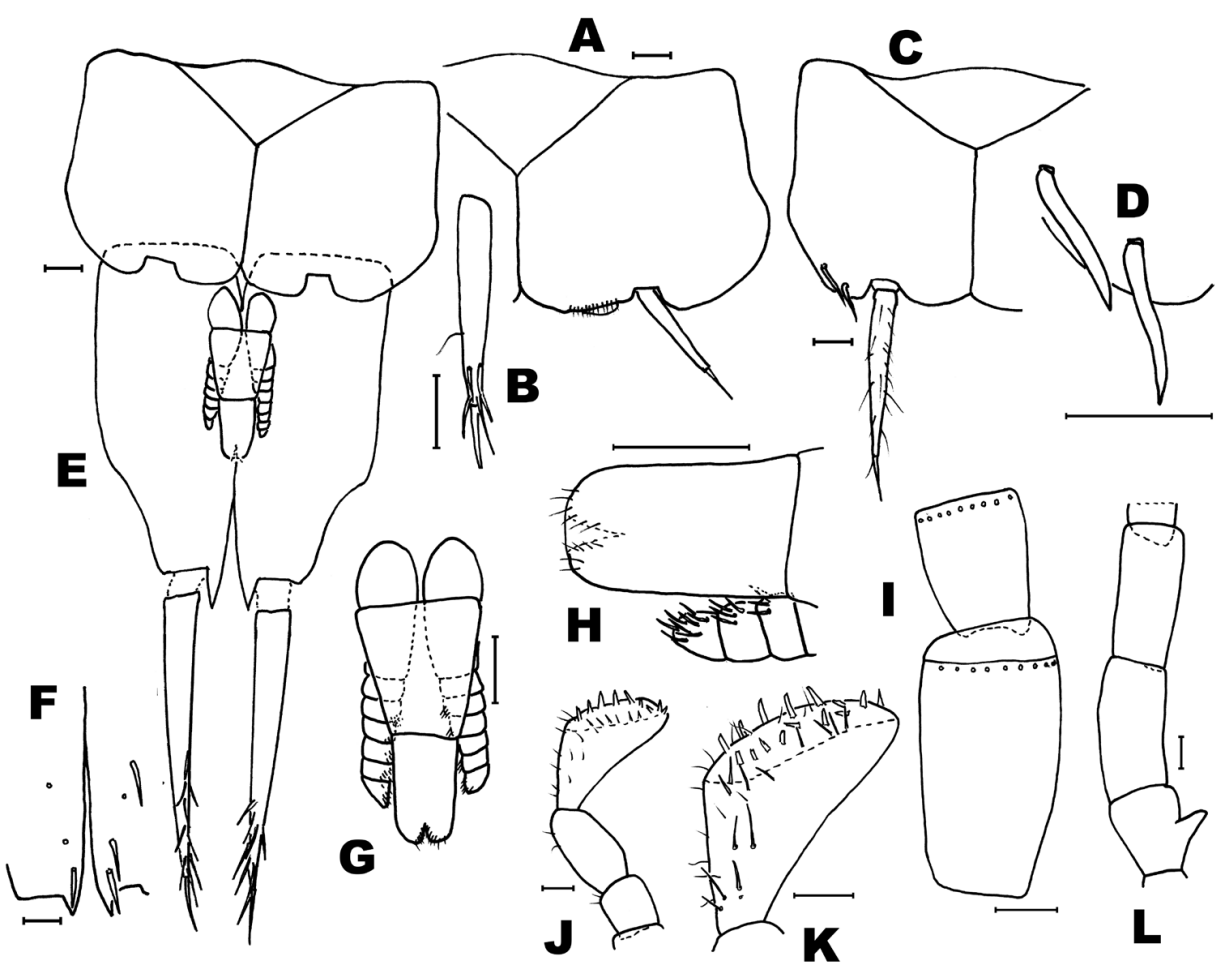
*Haslundiellairanica* sp. n.: male holotype (**A, B, E, G, H**), male paratype from Mirza cave (**C, D, F**) and female paratype (**I–L**). **A** fifth urosternite **B** fifth abdominal stylus **C** eighth urosternite **D** detail of lateral spines of eighth coxite **E** eighth and ninth urosternites with male genitalia **F** detail of spines in coxite IX **G** penis and nine parameres **H** penis opening **I** antennal scapus and pedicellus **J** labial palp **K** third labial palp article **L** preserved part of maxillary palp. Scale bar: 0.1 mm.

**Figure 6.**
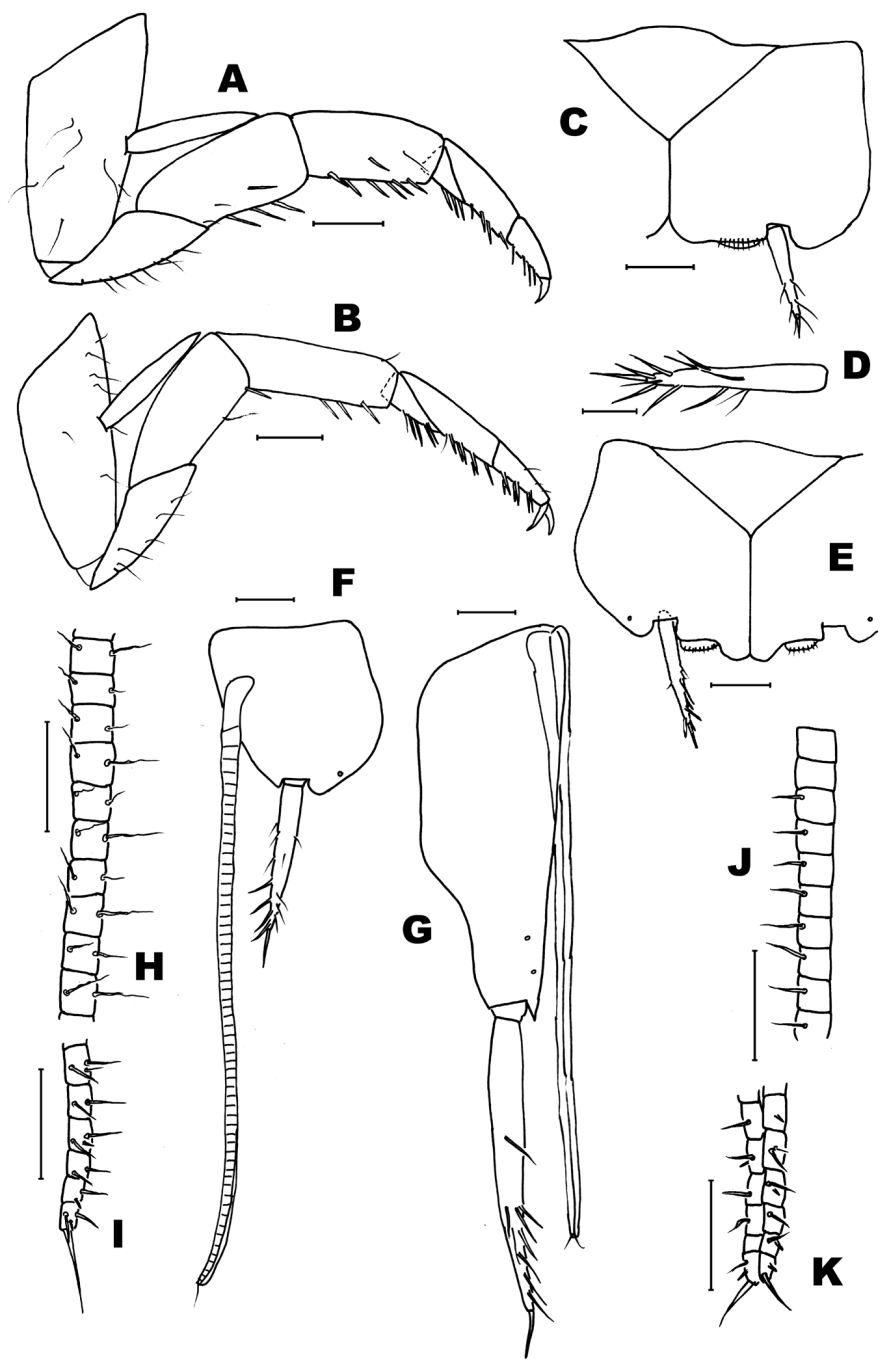
*Haslundiellairanica* sp. n.: female paratype. **A** mid leg **B** hind leg **C** fifth urosternite **D** fifth abdominal stylus **E** seventh urosternite **F** eighth urocoxite and gonapophysis **G** ninth urocoxite and gonapophysis **H** medial part of eighth gonapophysis (divisions 36–45) **I** distal part of eighth gonapophysis (divisions 53–58) **J** medial part of ninth gonapophysis (divisions 37–46) **K** distal part of ninth gonapophysis (divisions 53–58). Scale bars: 0.25 mm (**A–C, E**); 0.2 mm (**F, G**); 0.1 mm (**D, H–K**).

Figs 2 , 3 , 4 , 5 , 6 ; Tables 1 , 2

###### Type material.

**Holotype** (MNCN): male, body length = 8.5 mm, preserved in alcohol and partially mounted on slide, both vial and slide labelled “Mirza Cave, Rafsanjan, Kerman Province, Iran. 20.VI.2015. Cat. Types N. 2834”; “HOLOTYPE ♂ *Haslundiellairanica* sp. n., des. Gaju, Molero, Tahami & Sadeghi, 2018”. **Paratypes** (3 ex.): 1 male (UCO): same data as holotype, preserved in alcohol and partially mounted on slide, Ref. M1655a. 1 female juvenile, preserved in alcohol (Fig. 2): same locality and date, Ref. M1655b. 1 female (ZM-CBSU): from Khane Khoda cave, Heart, Yazd Province, Iran. 30.IX.2015, preserved in alcohol and partially mounted on slide (#C2636).

###### Diagnosis.

Machilidae of medium size, about 8–9 mm. Body unpigmented. Compound eyes wider than long (l/w = 0.65), lateral ocelli in sublateral position. Antennae shorter than body length, distal chains with a lower number of annuli compared with the remaining species of the genus. Coxal styli on middle and hind legs. Urosternites with 1+1 eversible vesicles; sternites with their posterior angle slightly obtuse, about 95°. Male without special chaetotaxy on maxillary palps and legs. Proximal part of the penis less than 1.3 times longer than the distal part. Female ovipositor with 1+58 divisions.

###### Description.

*Habitus of the new species*. All specimens, although coming from two different locations (about 300 km apart from each other), are very similar because their whitish, unpigmented bodies (Fig. 2). Body length of male 8.5 mm, female 9 mm. Body and appendages covered with scales and completely devoid of pigment. Paracercus and cerci broken. Dorsal scales pattern unknown. Meso and metathorax as usual in the order, not especially humped. Antennae shorter than the body, the specimen with longest antennae, a subadult female 7.5 mm long, with antennae of 3.9 mm (Fig. 2). Compound eyes black (Fig. 3, from adult female, those of males not clearly visible), wider than long (l/w: 0.65 and cl/l: 0.53). Frons not especially protruded between paired ocelli, which are brownish, sublateral, transversally ovoid: w/l: 2.29; not especially small (w.ocellus/w.eye: 0.55).

*Description of males.* Head: Antennae broken in the holotype (preserved length: 3.6 mm), scapus (Fig. 4A) not especially long (l/w: 1.52); distal chains with 10, 11 annuli (Fig. 4B); each annulus with one circle of bristles, slightly longer than the diameter of the annuli (Fig. 4C), some of them straight and strong others thinner and curled; the penultimate annuli of each chain with two specialized basiconic sensilla. Male maxillary palp not modified and without special chaetotaxy (Fig. 4D, E), all articles of similar length, only the third article slightly shorter (l 3^rd^/l 7^th^ = 0.88). Hyaline spines in articles 5, 6 and 7 typical, low in number (2, 6 and 7 respectively); article ratios shown in Table 1. Labial palp typical, third article not specially enlarged (Fig. 4F); sensorial cones typical, not very numerous (Fig. 4G), surrounded by strong bristles slightly longer than these cones (in the female this character is clearer).

Thorax: Second and third pair of legs with a stylus on the coxa, their length about 2/3 of the coxa (0.63). Fore femur not especially enlarged (Fig. 4H); hind tibiae longer than fore and mid tibiae (Figs 4H-J); all legs without special chaetotaxy, only with spines inserted ventrally on femora, tibiae and tarsi (Fig. 4K and Table 2); these spines have their distal part dark.

Abdomen: urosternites with medial sternite (Fig. 5A), slightly obtusangle (95°); with one pair of eversible vesicles on urocoxites I-VII; styli present on segments II-IX, the terminal spine of each stylus not so long; styli with strong subterminal spiniform setae (Fig. 5B); stylus/coxite and spine/stylus ratios shown in Table 1. Coxites VII-IX with hyaline spines (Fig. 5C, F and Table 1). Male genitalia without parameres VIII; penis and parameres IX completely covered by coxites IX, surpassing slightly half the coxite (Fig. 5E); parameres with 1+6–7 divisions (Fig. 5G), not surpassing the tip of penis; proximal part of penis longer than distal part, ratio pp/pd: 1.26. Penis opening subterminal (Fig. 5H).

**Table 1.** Comparison of several morphometric parameters of *Haslundiellairanica* sp. n. with the remaining species of the genus. **Length**: body length from frons until the end of tenth tergite; **eyes cl/l**: eye contact line/eye length; **eye l/w**: length/width of eye; **paired ocelli w/l**: width/length; **w ocelli/w eye**: width ocelli/width eye; **l ant**: length antennae; **scapus l/w**: scapus length/width; **ant d.c**.: number of annuli of antennal distal chains; **l cerci**: length cercus; **l p.cercus**: length paracercus; **mx-p7-p6-p5-p4-p3-p2/mx-p7**: length of maxillary palp articles 7–6–5–4–3–2/length of maxillary palp 7; **sp mx-p 7** and **sp mx-p 6**: spines on the dorsal side of maxillary palp articles 7^th^ and 6^th^; **l Ti1**: length of fore tibia; **Fe1 (l/w)**: length/width of fore femur; **l st-cx-L2-L3**: length of coxal stylus from second and third legs respectively; **l st/l cx-L2-L3**: length of coxal stylus/ length of coxa for second and third legs respectively; **angle of st abd V**: angle of urosternite V; **st/cx abd V, VIII, IX**: length stylus without spine/length coxite of urosternites V, VIII and IX; **sp/st abd V, VIII, IX**: length terminal spine/length of stylus without spine V, VIII and IX; **pp/pd penis**: length proximal /length distal part of penis; **parameres**: number of divisions of parameres; **g-VIII, IX**: number of divisions of gonapophysis VIII and IX. Data from *H.steinitzi* and *H.nisensis* obtained from text or drawings of original descriptions (those calculated from drawings are marked with bold).

	Males			Females		
	* H. steinitzi *	* H. nisensis *	*H.iranica* n. sp.	* H. steinitzi *	* H. nisensis *	*H.iranica* n. sp.
length (mm)	9.5	8.3–10	8.5		11–12	9
eyes cl/l	0.5	0.54–0.58			**0.5**	0.53
eyes l/w	0.7	0.74–0.77			**0.73**	0.65
paired ocelli w/l	**2.33**	2			**2.25**	2.29
w ocelli/w eye	**0.5**	0.57–0.59			**0.54**	0.55
l ant (mm)	> 9.5	> 8.3/10				3.6
scapus (l/w)			1.7			1.98
ant d.c.	18	13 (12–17)	10–11			
l cerci (mm)					4.9	
l p-cercus (mm)		>8.3–10				
mx-p 6/7	**1.54**	**1**	1	1.29		
mx-p 5/7	**2**	**1.42**	1.05	1.71		
mx-p 4/7	**2.23**	**1.46**	0.98	1.64		
mx-p 3/7	**0.77**	**0.88**	0.88	1		
mx-p 2/7	**1.31**	**0.88**	0.93	1.43		
sp mx-p 7		6–7	7		8–10	
sp mx-p 6		6–7	6		8–10	
l Ti1 (mm)			0.6			
Fe1 (l/w)	**2.17**	**2.26**	1.89			
l st-cx-L2-L3 (mm)		0.6–0.65	0.5			
l st/l cx-L2-L3		0.52–0.56	0.52			
angle st abd V	> 90°	95 °	95 °			95 °
st/cx abd V	0.4–0.45	0.55–0.65	0.43	0.4–0.45	0.55–0.65	0.45
sp/st abd V			0.32		**0.21**	0.32
st/cx abd VIII	0.9	1	0.7	0.9	1	0.87
sp/st abd VIII			0.27			0.27
st/cx abd IX	1	1.4	0.77	0.75	1	0.74
sp/st abd IX	**0.11**		0.21		**0.09**	0.15
pp/pd penis	**ca 1.87**	1.6	1.26			
parameres	1+(7–8)	1+7	1+(6–7)			
g-VIII				1+(45–50)		1+58
g-IX					1+65	1+58

*Description of females.* The only adult female has most of its appendages broken and some of them are lost, specifically right antennae, left maxillary palp, fore legs and left middle leg.

Head: As described in habitus section. Antennae broken, only the basal part of the left one is preserved; scapus similar to that of the male (Fig. 5I). Maxillary palp broken, the three basal articles preserved shown in Fig. 5L. Labial palp (Fig. 5J) as in male, but the strong setae surrounding the sensorial cones of the third article seem stronger (Fig. 5K).

Thorax: Middle and hind legs (Fig. 6A, B) similar to that of male, but slightly bigger; without special chaetotaxy, only ventral spines on femora, tibiae and tarsi (Table 2).

Abdomen: sternites typical of the genus, slightly obtusangle (95°), slightly bigger than those of male (Fig. 6C); styli of coxite similar to those of males (Fig. 6D). Coxite VII modified, with a terminal inner projection (Fig. 6E); one spine in its outer side. Coxites VIII and IX typical, with one spine in the outer side of the former (Fig. 6F) and two spines in the inner part of the later (Fig. 6G). Ovipositor of tertiary type, with 1 + 58 divisions, not attaining the apex of styli IX (Fig. 6G); gonapophysis VIII (Fig. 6H, I) with conspicuous chaetotaxy in the 22 distal divisions (36–58) and gonapophysis IX (Fig. 6J, K) in the 19 distal divisions (39–58), in the remaining divisions the chaetotaxy consists of very small bristles (if they are present).

**Table 2.** Comparison of the numbers of spines in different articles of legs of *Haslundiellairanica* sp. n. with the remaining species of the genus. **sp Fe1, 2, 3**: number of spines on fore, mid and hind femur; **sp Ti1, 2, 3**: number of spines on fore, mid and hind tibia; **sp Ta1–1, 2, 3**: number of spines on the three articles of fore tarsus (the same for mind tarsus –**sp-Ta2**- and hind tarsus –**sp-Ta3**).

	Males			Females		
	* H. steinitzi *	* H. nisensis *	*H.iranica* n. sp.	* H. steinitzi *	* H. nisensis *	*H.iranica* n. sp.
sp Fe1	1	1	1			
sp Ti1	2	3–6	2–4			
sp Ta1–1		3–6	2–3			
sp Ta1–2		8–12	6–7			
sp Ta1–3		6–8	6			
sp Fe2	1		1–2			
sp Ti2	2		3–6			6
sp Ta2–1			2–4			3–4
sp Ta2–2			5–6			8
sp Ta2–3			6			6
sp Fe3	1		0–1			0–2
sp Ti3	5		3–4			7
sp Ta3–1			4			6
sp Ta3–2			7			8–9
sp Ta3–3			6			5–6

###### Discussion.

The first species described of the genus *Haslundiella* Janetschek, 1954 was found in Palestine and was named as *Praemachilissteinitzi* (Wygodzinsky, 1942). In their description Wygodzinsky (1942) suggested that “*The remarkable formation of maxillary palp and the genitalia of the male might, in the future, lead to establish a new genus within the Praemachilinae*”. Janetschek (1954) erected the genus *Haslundiella*, including in it only *H.steinitzi*. Kaplin (1982) described the second species of this genus (*H.nisensis* Kaplin, 1982) from Turkmenistan. Now, *Haslundiellairanica* sp. n. is described from Iran, geographically placed between the two former species.

*Haslundiellairanica* sp. n., can be distinguished from the other species by several characters, with the most remarkable being the significant absence of special chaetotaxy on the maxillary palps and the legs of males. Moreover, some other characters can be mentioned: Antennae are shorter than body length; the shape of the maxillary palp is different because all articles are similar in length, showing clear differences with *H.steinitzi* and *H.nisensis* (Table 1); and legs are similar in shape but with very different chaetotaxy. The male genitalia of *H.iranica* is clearly different from that of *H.steinitzi* and similar to *H.nisensis*, although penis ratios are different to those of both previously described species. The female ovipositor does not surpass the styli IX, as in *H.nisensis*, although with lower number of divisions, meanwhile that of *H.steinitzi* has a low number of divisions but surpasses the styli IX.

###### Distribution.

Only known from the two localities in Kerman and Yazd provinces in Iran (see Fig. 1).

###### Etymology.

The name *iranica* is a genitive case of the name Iran, the country where the new species is found.

###### Habitat.

The specimens were found and collected close to the entrance of the cave where it was still darker and more humid than the outside (somewhere between the endogean and parahypogean zones) and couldn’t be found deeper in as the cave is a complex of horizontal (about 50 m from the entrance) and then vertical (a 50 m of a vertical pit) passages reaching a big hall at the end.

##### Key to *Haslundiella* species

**Table:** 

1	Males	**2**
–	Females	**4**
2	Maxillary palp with especial chaetotaxy	**3**
–	Maxillary palp without especial chaetotaxy	***H.iranica* sp. n.**
3	Fore femur and tibia with short delicate setae; tibia with a dorsal field of long bristles	*** H. steinitzi ***
–	Fore femur without especial chaetotaxy	*** H. nisensis ***
4	Ovipositor projecting beyond styli IX for one third (with 45–50 divisions); ratio stylus/coxite IX: 0.75	*** H. steinitzi ***
–	Ovipositor not surpassing the IX styli	**5**
5	Ovipositor with 65 divisions; ratio stylus/coxite IX: 1	*** H. nisensis ***
–	Ovipositor with 58 divisions; ratio stylus/coxite IX: 0.74	***H.iranica* sp. n.**

### Order Zygentoma

#### Family Lepismatidae

##### Ctenolepisma subterraneum

Taxon classificationAnimaliaZygentomaLepismatidae

Molero, Tahami, Sadeghi & Gaju sp. n.

http://zoobank.org/08CC79A7-2E2C-4330-A6C8-4028B5503B47

Figs 7 , 8

###### Type material.

**Holotype** (MNCN): female, body length = 7 mm, mounted on slide, labelled “Abu Nasr cave, Shiraz, Fars Province, Iran. 12.XI.2015. Cat. Types N. 2835”; “HOLOTYPE ♀ *Ctenolepismasubterraneum* sp. n., des. Molero, Tahami, Sadeghi & Gaju, 2018”.

###### Diagnosis.

Very faintly pigmented and medium-sized lepismatid. Distribution of scales and trichobothrial areas as in *C.ciliatum*. Apical article of the labial palp with almost parallel sides and three sensory papillae arranged in a single row. Pronotum with 9–10 combs of macrosetae, mesonotum with 14 pairs and metanotum with 11 pairs of combs. Prosternum with 2+2 bristle-combs, mesosternum with 1–2 pairs of combs and metasternum with 1+1 combs. Macrosetae of thoracic sternites arranged in one row. Urosternites III-VIII with 1+1 lateral combs. All urosternites without median combs (subgenus Ctenolepisma s. str. sensu Irish, 1987). Urotergite I with 1+1 combs, II-VI with 3+3 combs and VII-VIII with 2+2 combs (*ciliatum*-group sensu Mendes, 1982). Urotergite X trapezoidal. Two pairs of styli. Ovipositor with 40 divisions. Male sex unknown.

###### Description

**Figure 7.**
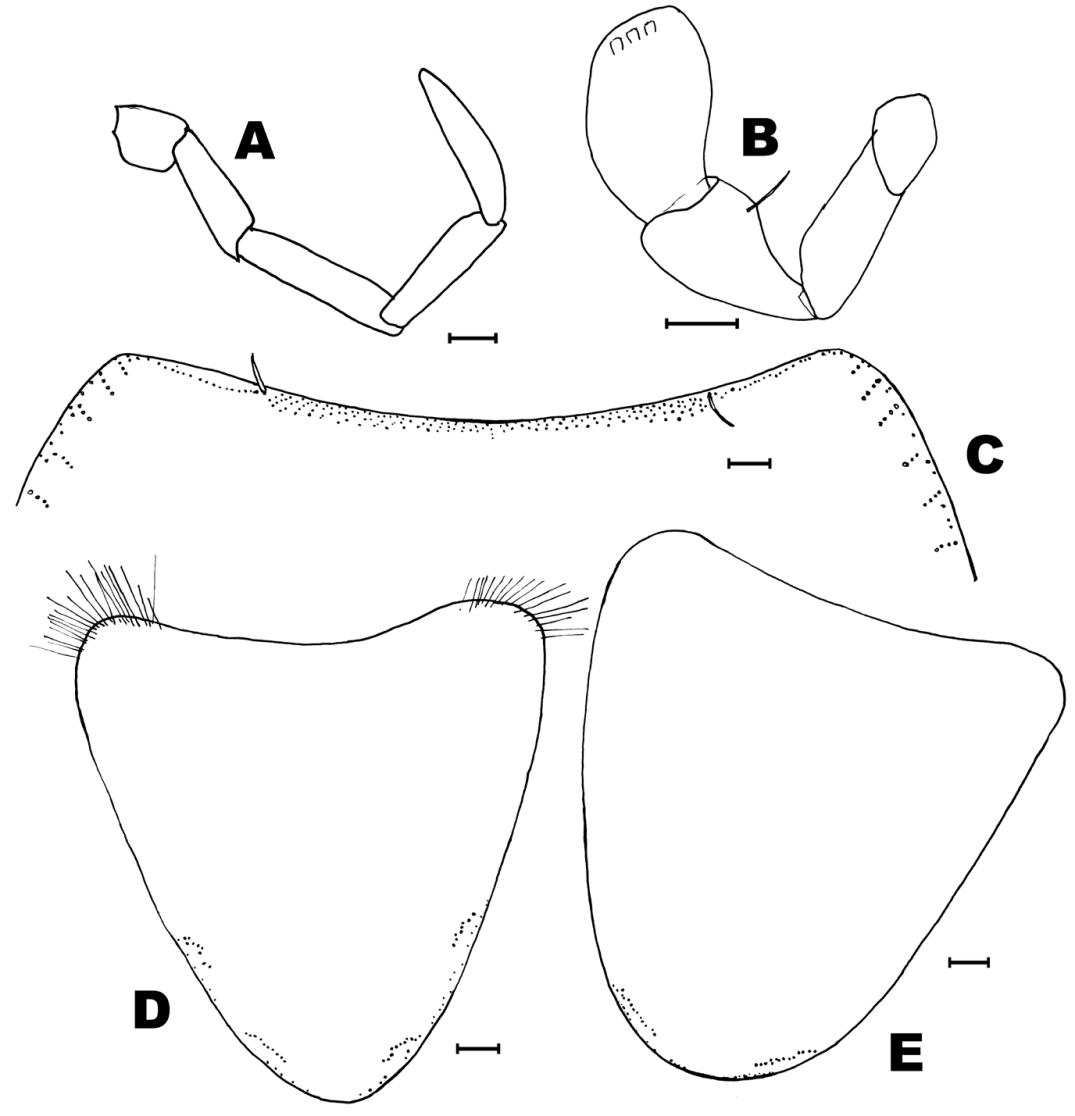
*Ctenolepismasubterraneum* sp. n. holotype, head and thorax. **A** maxillary palp **B** labial palp **C** anterior part of the pronotum, showing insertions of macrosetae **D** prosternum **E** mesosternum. Scale bar: 0.1 mm.

. Body length: 7 mm. Body fusiform, with the thorax slightly wider than the abdomen, maximum thorax width 1.75 mm. Epidermal pigment very faint, yellowish-brown in alcohol. Scales with brownish pigment (perhaps greyish in live specimens). Most macrosetae are lost, they can be detected by their insertions, when preserved they are almost hyaline to brown-yellowish. Setation of the head with the pattern typical of the genus. Eyes with 12 ommatidia. Antennae broken (maximum length preserved: 2.2 mm). Maxillary palp (Fig. 7A) with the apical article about 5.5 times longer than wide and as long as the penultimate. Apical article of the labial palp about 1.4 times longer than wide, without any expansion in the inner side, with three sensory papillae arranged in a single row (Fig. 7B). Pronotum with a brush of macrosetae in the central part of the anterior margin, with 2–4 rows of macrosetae. A row of 20–24 short smooth setae extends from the median brush to the anterolateral corner of the pronotum (Fig. 7C). The lateral margins of this notum show 9+10 lateral combs with 2–5 macrosetae each. Mesonotum with 14+14 lateral combs with 1–5 macrosetae (the two anterior “combs” are composed only by one macroseta). Metanotum with 11+11 lateral combs with 1–4 macrosetae (the first composed also by one macroseta). Additionally, the three nota have 1+1 posterolateral combs with 6–8 macrosetae. Anterior trichobothrial areas of the thoracic nota not well visible, but apparently following the same arrangement than in *Ctenolepismaciliatum*, i.e., those of the pronotum situated on lateral comb N-3; those of the mesonotum associated with the antepenultimate (N-2) lateral comb and those of the metanotum associated with the penultimate (N-1) lateral comb. All posterior trichobothria associated with the last lateral comb (N) on the three nota.

**Figure 8.**
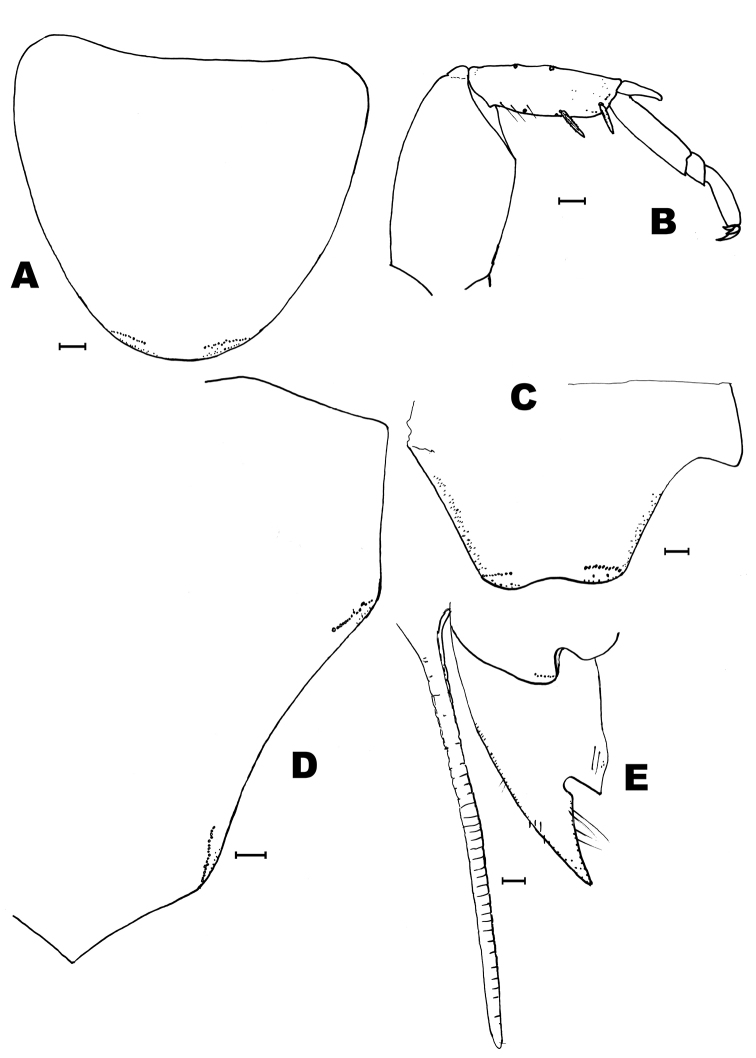
*Ctenolepismasubterraneum* sp. n. holotype, thorax and abdomen. **A** metasternum **B** last articles of middle leg, showing macrochaetae and their insertion on the tibia **C** urotergite X **D** urosternite VII **E** hind margin of coxite VIII, coxite IX and ovipositor (styli VIII and IX lost). Scale bar: 0.1 mm.

Prosternum heart shaped, as long as wide at its base and with the hind margin rounded but slightly truncated at the apex (Fig. 7D). It bears 1+1 brushes of thin and long setae in the anterolateral corners and 2+2 oblique combs in the antedistal region; each comb with 11–12 macrosetae arranged in a single row, although the anterior ones are more irregular. Mesosternum slightly longer than wide at its base, with only 1+1 oblique antedistal combs of 11–14 macrosetae arranged in a single row (Fig. 7E), although the comb of the left side is irregular, apparently broken in two close smaller combs (could be interpreted as 1+2 combs). Metasternum wider than long (ratio length/ width about 0.85) with only one pair of combs of 13–14 macrosetae arranged in one row (Fig. 8A). Hind margins of meso- and metasternum slighly truncated. Distance between the combs 1.4–1.5 times the width of a comb. Protibiae 2.9–3.0 times longer than wide; mesotibiae 3.2 times longer than wide and about 1.15 times longer than the protibiae; metatibiae lost. All preserved tibiae with 2 dorsal and 4 ventral macrosetae shorter than diameter of the article (Fig. 8B). Tibiae without scales. Scales of femora rounded.

Urotergite I with 1+1 bristle-combs; urotergites II-VI with 3+3 combs; urotergites VII and VIII with 2+2 combs. Submedian bristle-combs with 6 macrosetae, lateral combs with 6–7, and sublateral combs with 7–10. Urotergite X trapezoidal, somewhat long (its apical part about 0.46 longer than wide at its base), with slightly concave posterior margin and 1+1 combs of 10–11 macrosetae (Fig. 8C). Urosternites I and II without setae, III-VIII with 1+1 lateral bristle-combs with 11–15 macrosetae. Distance between lateral combs of urosternites 4.8–5.6 times wider than the width of a comb (Fig. 8D).

Male sex unknown. In female, two pairs of abdominal styli (they are lost, but their insertions are clearly visible). Inner process of coxite IX about 1.8 times longer than wide at its base and 3.1 times longer than the outer process (Fig. 8E). Ovipositor with 40 divisions, its apex surpassing the tip of the inner process of the coxite IX approximately by its length (Fig. 8E). Apices of gonapophyses unsclerotized. Caudal filaments broken; maximum length preserved 1.3 mm (in a cercus).

###### Discussion.

The new species is related to *C.ciliatum* (Dufour, 1831), *C.longicaudatum* Escherich, 1905 and *C.armeniacum* Molero, Gaju, Bach & Mendes, 2010, sharing with them the following characteristics: abdominal setation (absence of median combs on urosternites, 3+3 combs of macrosetae on urotergites II-VI), the trapezoidal shape of the tenth urotergite, the distribution of scales on legs (rounded on femora and absent on tibiae and tarsi), the distribution of trichobothria of the nota, and the smooth setae of the anterolateral row of the pronotum. However, all of these species show 5 papillae in the apical article of the labial palp, whilst *Ctenolepismasubterraneum* sp. n. has 3 papillae. There is only one species of the genus *Ctenolepisma* that shares this particular characteristic in the labial palp and the aforementioned abdominal characters: *C.barchanicum* Kaplin, 1985, from the Karakum region in Turkmenistan, but this species is clearly a different taxon because several differences can be detected:

– Number of pairs of styli: *C.barchanicum* has only one pair of styli, and *C.subterraneum* sp. n. has two pairs (at least, females);

– Shape and setation of thoracic sternites: Kaplin’s drawings of *C.barchanicum* reveal that these sternites are clearly rounded at their apex, while in the new species their hind margins are slightly truncated. Moreover, the number of combs in the Turkmenian species is higher (5–8 pairs in the prosternum, 3–4 pairs in the mesosternum and 2 pairs in the metasternum, versus 2, 1–2 and 1 pairs of combs respectively in the Iranian species);

– Shape of the apex of the labial palp: Following Kaplin (1985), the inner side of the apical article of the labial palp is strongly widened in *C.barchanicum*, while in *C.subterraneum* sp. n. the labial palp has subparallel sides;

– Number of lateral combs in nota: According Kaplin’s description (in Russian), the Turkmenian species has a lower number of combs in the pronotum (5 pairs), in the mesonotum (8–10 pairs) and in the metanotum (7–9 pairs), while the new species from Iran has respectively 9+10 combs, 14 pairs and 11 pairs of combs;

– Shape of the hind margin of the urotergite X: Kaplin’s drawing of the urotergite X of *C.barchanicum* shows a convex hind margin, while this is concave in *C.subterraneum* sp. n.;

– Length and number of divisions of the ovipositor. *C.barchanicum* has only 12–13 division and the new species has 40 divisions. In spite of this, the length of the ovipositor of *C.subterraneum* sp. n. is only slightly higher relative to coxites IX.

The genus *Ctenolepisma* was revised recently in Iran by Kahrarian et al. (2016); then, seven species were considered to occur in this country, if *C.mauritanicum* (Lucas, 1846), with doubtful status, is included. *Ctenolepismasubterraneum* sp. n. fits at step 5 of the key of Kahrarian et al. (op. cit.) with the following modifications:

**Table:** 

5	Macrosetae in meso and metasternum arranged in combs of 2 or 3 irregular rows	**6**
–	Macrosetae in meso and metasterum arranged in combs of 1 row	**5**’
5’	Apical article of the labial palp with 5 papillae. Prosternum with acute apex and usually with 3 or more pairs of bristle-combs. Combs of urotergites with more than 8 macrosetae	***C.ciliatum* (Dufour, 1831)**
–	Apical article of the labial palp with 3 papillae. Prosternum with rounded and slightly truncated apex and with only 2 pairs of bristle-combs. Number of macrosetae of the submedian and lateral combs of urotergites lower than 8	***C.subterraneum* sp. n.**

###### Distribution.

Known only from the type locality, Abu Nasr cave, in Fars province, Iran.

###### Etymology.

The specific name of the new species refers to its habitat, not common within species of the genus (see next section). Subterraneum is an adjective in the nominative case.

###### Habitat.

This new species has been found near the cave´s entrance (endogean). This habitat is unusual within members of the genus *Ctenolepisma*, since most of them are associated to more superficial (epigean) habitats: under stones or vegetal debris, in trunks of trees, etc.

##### Acrotelsa collaris

Taxon classificationAnimaliaZygentomaLepismatidae

(Fabricius, 1793)

###### Studied material.

Two young specimens from Khan cave, Khon, Fars province, Iran. 10.IX.2015, one deposited in UCO, Ref. Z2517, and the other in ZM-CBSU #C2637.

###### Distribution and habitat.

This pantropical species has been previously reported from Iran (Irish 1995, Kahrarian et al. 2014), but never in a subterranean habitat. The two specimens were collected in the dark zone of the cave (hypogean), where humidity is greater than 90% and the soil was wet, mixed with piles of guano.

##### Neoasterolepisma palmonii

Taxon classificationAnimaliaZygentomaLepismatidae

(Wygodzinsky, 1942)

###### Studied material.

One male, mounted in a slide. Abu Nasr cave, Shiraz Province, Iran. 12.XI.2015. Deposited in UCO, Ref. Z2515.

###### Distribution and habitat.

This species is new for Iran. It was previously known from Turkey and Israel (Mendes 1988), so this record represents a significant extension of its geographic distribution. The only specimen available has been collected from the endogean zone of the cave. The only species of this genus recorded previously from a cave is *N.caeca* Molero-Baltanás, Bach de Roca & Gaju-Ricart, 1999, collected inside a lava cave (Molero et al. 1999) in La Palma (Canary Islands).

##### Ctenolepisma (Sceletolepisma) targionii

Taxon classificationAnimaliaZygentomaLepismatidae

(Grassi & Rovelli, 1889)

###### Studied material.

One male, mounted in a slide, two females and one juvenile in alcohol. Endogean zone in Dej Shapour cave, Kazerun, Fars Province, Iran. 21.X.2015. Deposited in UCO, Ref. Z2516.

###### Distribution and habitat.

This species is widespread in the southern Palaearctic, but it is new for Iran. It is the first time that it has been recorded in the subterranean environment. *Ctenolepismatargionii* is found as a domestic form in the Western Palaearctic, but it lives in natural habitats in southwestern Asia, suggesting that it is native from the latter area.

###### Discussion.

Together with the above-described *Ctenolepismasubterraneum*, the number of species of this genus in Iran increases to nine; *C.targionii* represents the third species known for Iran of the subgenus Sceletolepisma.

#### Family Nicoletiidae

##### Lepidospora (Brinckina) momtaziana

Taxon classificationAnimaliaZygentomaLepismatidae

Molero, Tahami, Sadeghi & Gaju sp. n.

http://zoobank.org/2E74DC29-44F0-4DDB-9C28-B833420D9F89

**Figure 9.**
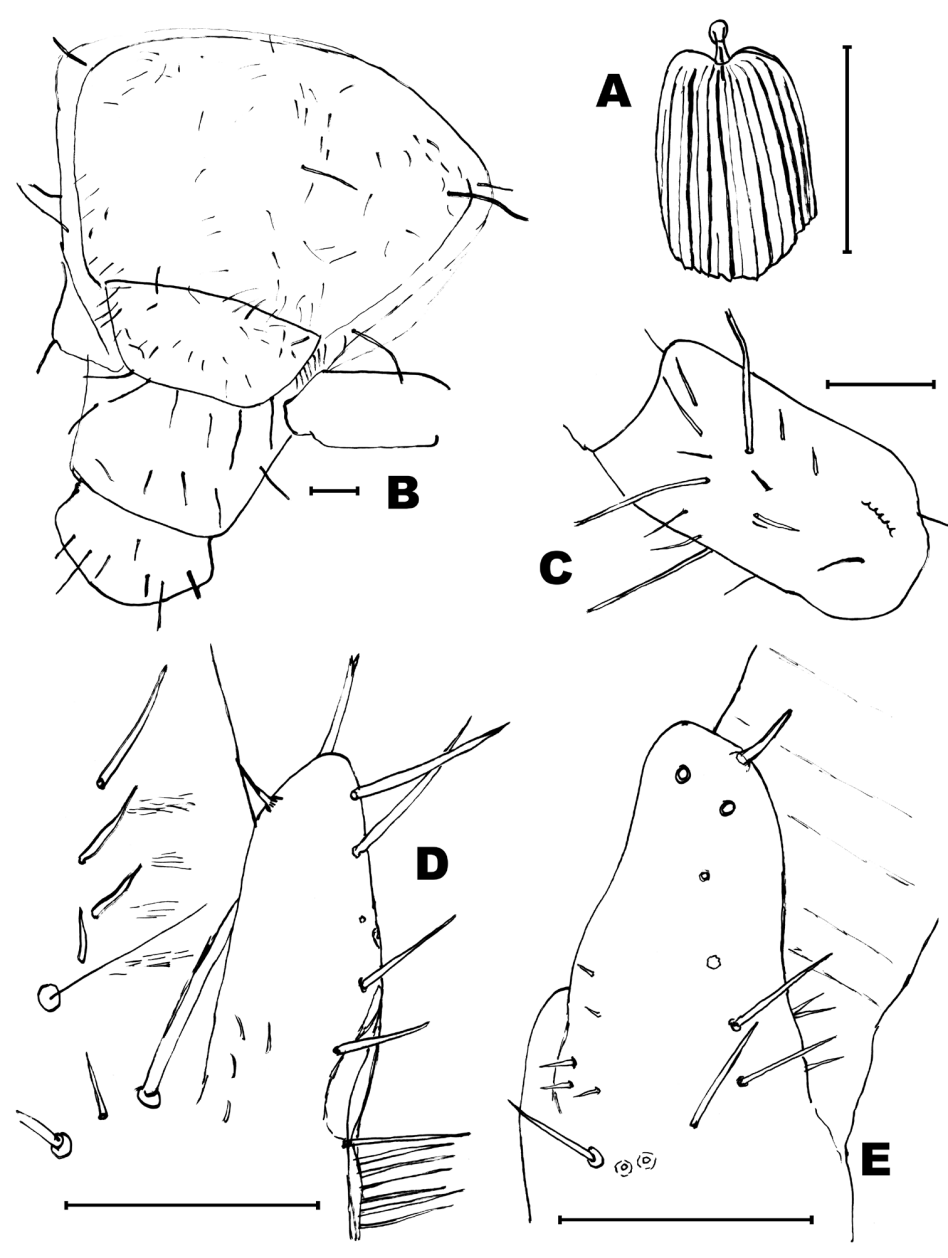
*Lepidosporamomtaziana* sp. n. **A** abdominal scale **B** head **C** scape **D** apophysis of the right pedicel of the male **E** apophysis of the left pedicel of the male. Scale bars: 0.1 mm (**B–E**); 50 μm (**A**).

**Figure 10.**
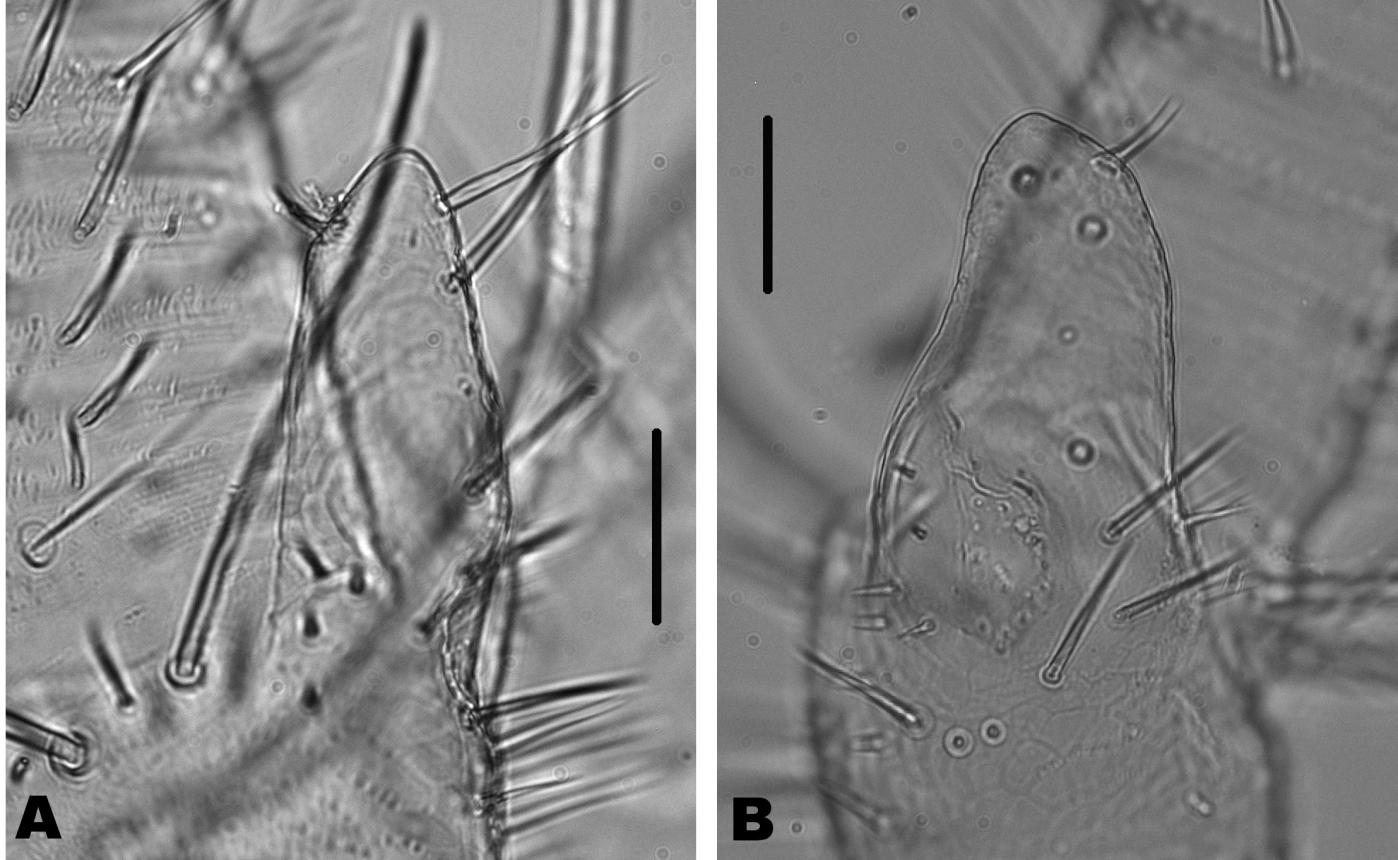
*Lepidosporamomtaziana* sp. n. micrographs of the pedicelar apophyses of the male (holotype). **A** right apophysis **B** left apophysis. Scale bar: 0.1 mm.

**Figure 11.**
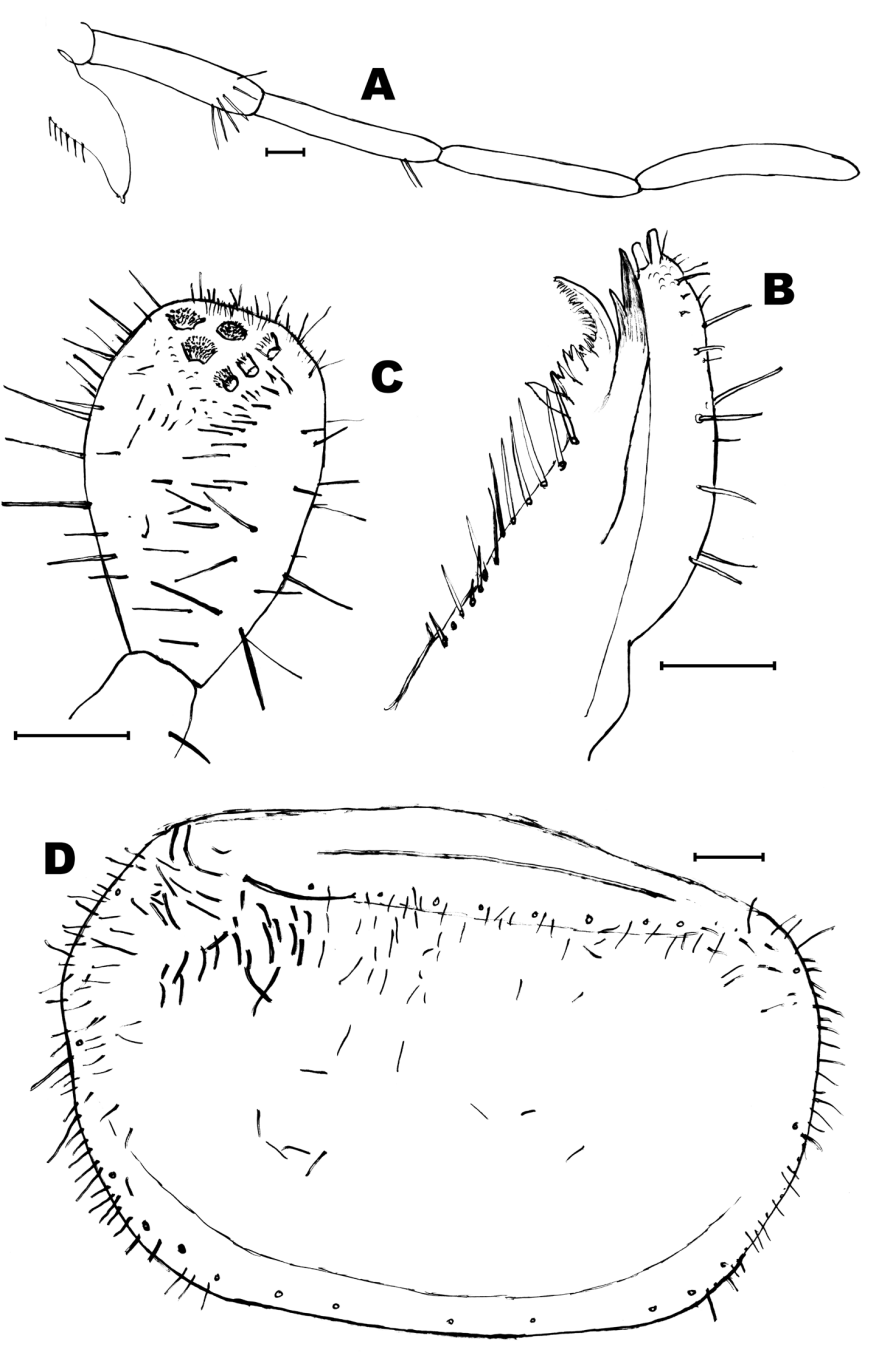
*Lepidosporamomtaziana* sp. n. **A** maxillary palp **B** apex of maxilla **C** apical article of the labial palp **D** pronotum (only few macrosetae are preserved but their insertions are indicated). Scale bar: 0.1 mm.

**Figure 12.**
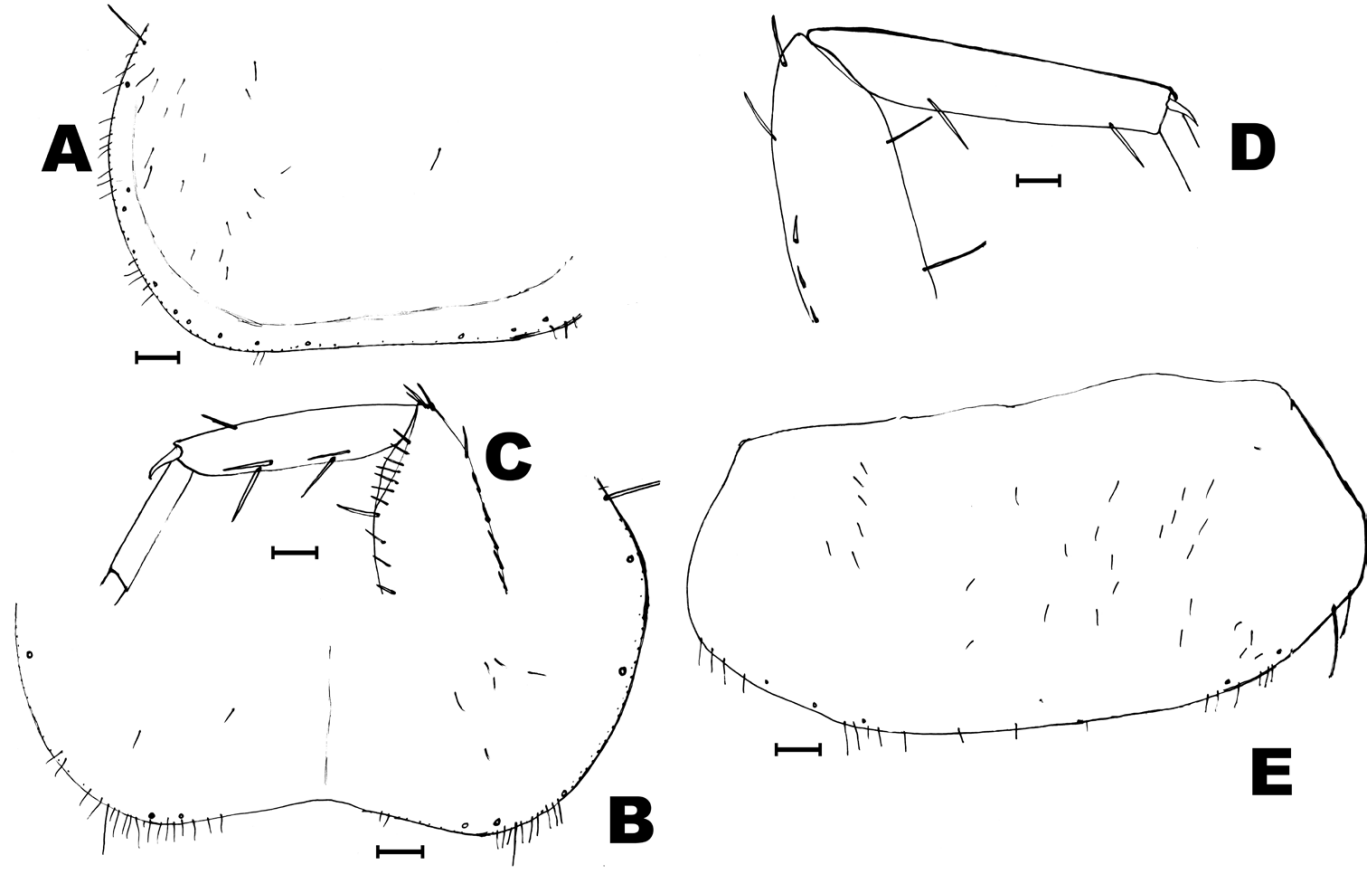
*Lepidosporamomtaziana* sp. n. **A** mesonotum, lateral and hind margin **B** metanotum, lateral and hind margin **C** protibia **D** metatibia **E** urotergite I. Scale bar: 0.1 mm.

**Figure 13.**
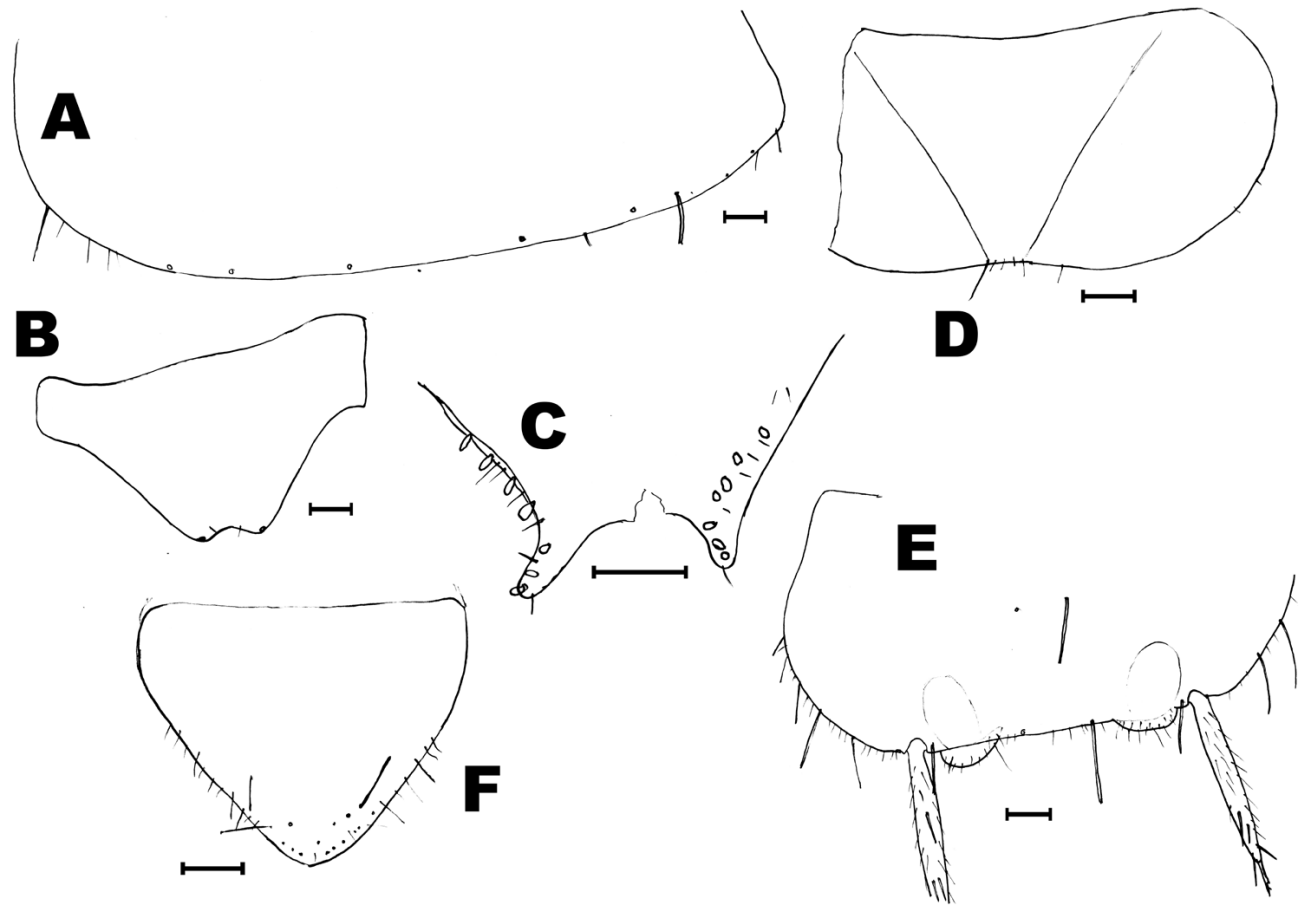
*Lepidosporamomtaziana* sp. n., abdomen. **A** urotergite V **B** urotergite X of the female **C** urotergite X of the male, hind margin showing pegs **D** urosternite I **E** urosternite V **F** subgenital plate of the female. Scale bar: 0.1 mm.

**Figure 14.**
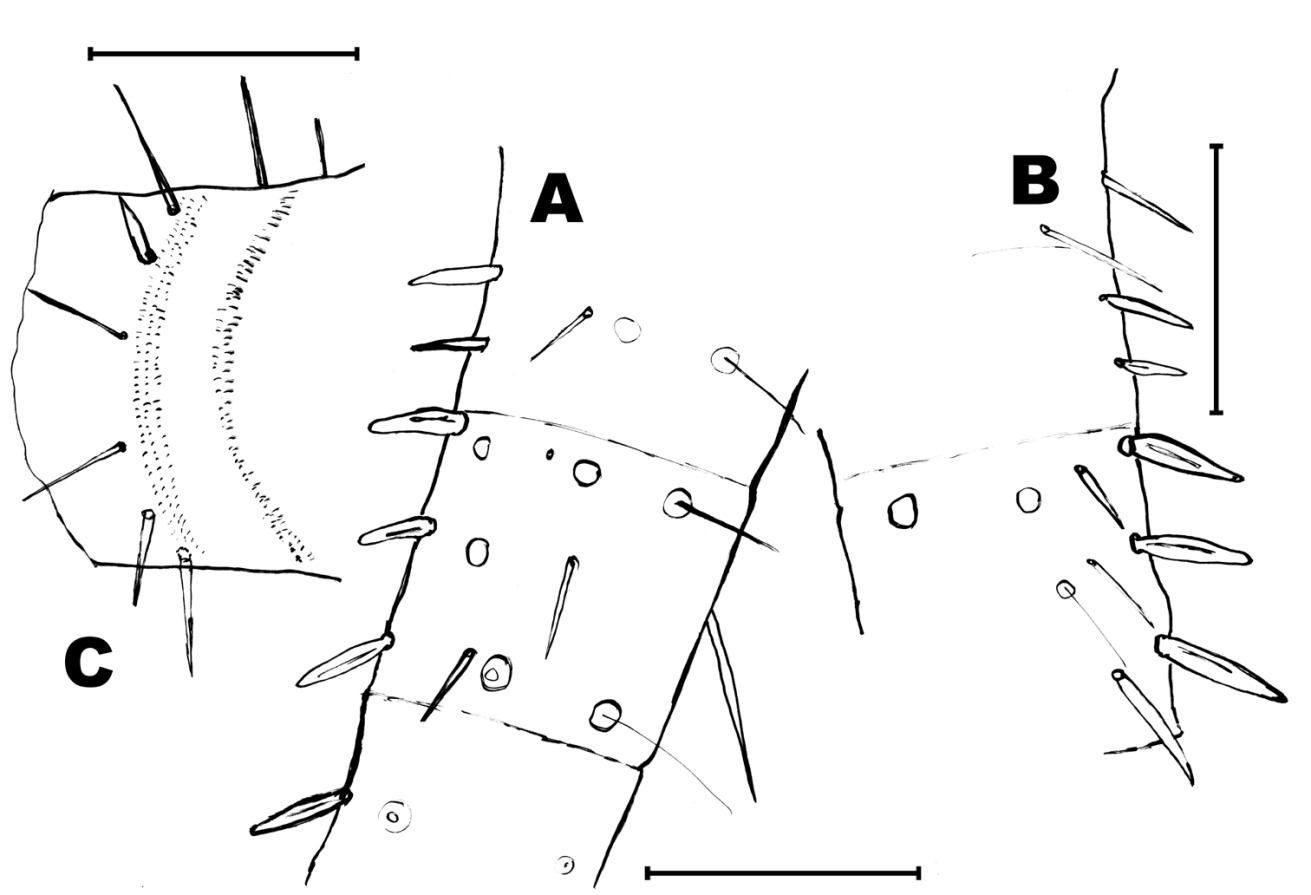
*Lepidosporamomtaziana* sp. n., holotype, terminal filaments. **A** basal part of the right cercus. **B** inner margin of the basal part of the left cercus **C** basal portion (only preserved) of appendix dorsalis (=paracercus). Scale bar: 0.1 mm.

Figs 9 , 10 , 11 , 12 , 13 , 14 , Table 3

###### Type material.

**Holotype** (MNCN): male, body length = 7 mm, mounted on slide, labelled “Momtaz cave, Marvdahst, Fars Province, Iran. 11-XI-2016, Cat. Types N. 2836”; “HOLOTYPE ♂ Lepidospora (Brinckina) momtaziana sp. n., des. Molero, Tahami, Sadeghi & Gaju, 2018”. **Paratypes** (2 ex.): One female, collected in the same locality and date (preserved in alcohol and deposited in ZM-CBSU #C2638). One female from the same locality, 18-II-2015, mounted in slide and reported as Lepidospora (Brinckina) sp. in Tahami et al. (2018), deposited in UCO (Ref. Z2513).

**Table 3.** Comparing Lepidospora (Brinckina) momtaziana sp. n. with other previously described species of the subgenus Brinckina.

SPECIES (geographic origin)	Shape (and ratio length/width) of pedicellar apophysis	Chaetotaxy of the discs of nota	Number and distribution of pegs in cerci
L. (B.) momtaziana sp. n. (Iran)	Asymmetrical, left thumb-like (1.6) and right subcylindrical (2.4)	Sparse and short microchaetae	6 in a single row
			Proximal division: 1–2
			Second division: 3
			Third division: 0–1
L. (B.) relicta (Australia)	Symmetrical, subcylindrical (about 3)	Sparse short setulae	4 in a single row
			Proximal division: 0
			Second division: 2
			Third division: 2
L. (B.) garambensis (Congo)	Symmetrical, subcylindrical (about 3.8) but somewhat curved	Sparse long setae	3–6 in a single row
			Proximal division: 0–1
			Second division:2–3
			Third division: 1–2
L. (B.) hamata (Congo)	Symmetrical, subcylindrical (about 1.7)	Dense setae	4 in a single row
			Proximal division: 0
			Second division: 3
			Third division: 1
L. (B.) alticola (Kenya)	Males unknown	Moderately dense and long setae	Males unknown
L. (B.) hemitrichoides (Afghanistan)	Symmetrical, subcylindrical with acute apex (about 2.5?)	Dense setae	Proximal division: 5? 2?
			Second division: 1? 3?
			Third division: 0? 1? *
L. (B.) makapaan (South Africa, Transvaal)	Symmetrical, subconical, curved apically (about 2.3)	Few isolated microchaetae	0
L. (B.) hemitricha (China, Vietnam)	Symmetrical, subcylindrical, slightly narrowing towards apex (about 2.5)	Dense and long setae, very dense in prothorax	5 spiniform in a single row
			Proximal division: 1
			Second division: 2
			Third division: 2

*There are two ways of interpreting Wygodzinsky’s drawings of cerci: the first implies that he did not draw a limit between the proximal and second division but that the limit exists (sometimes it is difficult to discern) in which case the arrangement of pegs would be 2–3–1 as in the new species from Iran; secondly, the aforementioned limit does not exist and, in this case, the arrangement would be 5–1.

###### Diagnosis.

Light yellowish nicoletiid; adults about 7 mm long, antennae slightly longer. Body covered with scales except on head (typical in the subgenus Brinckina). Pedicel of male antennae with slightly asymmetrical apophyses; this asymmetry involves shape and chaetotaxy. Dorsally with few setae, those inserted on the disc of nota very small and sparse, their length about 1/20 of the respective notum. Male urotergite X with 7+7 pegs, 3+3 of them inserted on the posterolateral projections. Subgenital plate of female widely triangular. Male cerci with 6 pegs arranged in a single row, the basal division with 1–2 pegs.

###### Description

. Body length of the male (holotype): 6.8 mm. Length of the female (paratype): 7.7 mm.

Thorax length: 2.5–2.6 mm. Thorax width: 1.4–1.7 mm. Shape of the body subcylindrical, the thorax nearly as wide as the abdomen. Epidermal pigment light yellowish, slightly darkened in the abdomen; gut contents are visible because transparency of the teguments. Head completely devoid of scales, thorax and abdomen covered dorsally and ventrally by scales. Scales as in Figure 9A, a little longer than wide, thoracic scales about 40–50 µm long, with 6–8 rays which extend slightly beyond the margin, abdominal scales slightly larger, with 8–15 rays.

Head prognathous, with some bifid macrosetae inserted in the lateral margins of the cephalic capsule, frons and in the middle of clypeus and labrum. Some dispersed setae are irregularly arranged in the cephalic capsule (Fig. 9B).

Antennae slightly longer than body; in the holotype they are 7.6 mm long. Scape 1.5 times longer than wide and almost twice longer than pedicel (3 times longer in the female), with 3 bifid macrosetae inserted on its apical half, and some additional thin setae of variable length (Fig. 9C). Pedicels of the male with apophyses that appear to be asymmetric. They are subcylindrical but the left apophysis is broader basally and narrower in its apical half (ratio length/width at the base: 1.6), with a blunt apex and the right apophysis is slightly longer (ratio length/width at the base: 2.4), without abrupt narrowing in its distal half and apically more acute. Both apophyses are similar in length (their distal end reaches the level of the third annuli); so it can be thought that this different shape could be explained by a different angle of vision because their different position in the slide, but the chaetotaxy of both apophyses does not match; the left apophysis has two insertions of setae nearly at the same level in its apical part and the right apophysis shows two acute setae in the apical part but they are inserted in different positions. Both apophyses have a glandular seta inserted subapically and the right apophysis has a more prolonged apex beyond the insertion of the seta. In the basal part of the apophyses, there is a fovea with several small setae. Three additional long macrosetae are inserted in the distal part of the trunk of the pedicel, just under the limit with the flagellum (compare Figs 9D and 10A with Figs 9E and 10B). Pedicel of the female without apophysis and with five long macrosetae. Basiconic sensilla long, abundant on the flagellum, especially in T-joints, i.e., those annuli bearing trichobothria. Mandibles and maxillae without distinctive features. Last article of maxillary palps only preserved in the holotype, with several (usually 5) apical sensory rods and a subcircular sensilla apically. The three distal articles of these palps (last, penultimate and antepenultimate) with scattered long basiconic sensillae. Apical article of the maxillary palp about 6.8 times longer than wide and 1.15 times longer than the penultimate (Fig. 11A); this latter of similar length than the antepenultimate. Galea with two apical conules (Fig. 11B). Apical article of the labial palp about 1.5–1.7 times longer than wide and 1.5–1.6 times longer than the penultimate, with 6 sensory papillae arranged as usual in the genus (Fig. 11C). Inner side of this article with 5–6 thin-walled basiconic sensilla; outer side with 4–5 similar sensilla, most slightly curved basally and inserted in the basal half of the article.

Most thoracic and abdominal macrosetae are lost in both available specimens and only their insertions are visible; when preserved, their length is about 1/4 – 1/5 of the length of the respective tergite. Nota (Figs 11D, 12A, B) with several bifid macrosetae of variable length irregularly inserted on their lateral and posterior borders; the pronotum also bears these setae (10–14) on its anterior margin, although most of them are lost and only their insertions are visible (Fig. 11D). Moreover, there are a lot of small simple setae over the lateral margins of the nota and in the anterior margin of the pronotum and the posterior margin of the metanotum (Fig. 12B). These thin and short setae (considered as microchaetae) are very scarce in the disc of the nota but there is a significantly higher number in the anterior part of the pronotum of the holotype (Fig. 11D).

Protibiae about 4.2–4.4 times longer than wide, with 2 dorsal and 4–5 ventral spines (apart from a row of 5–7 short spines in the ventro-apical angle of the tibiae (Fig. 12C). Mesotibiae about 4.5–4.75 times longer than wide, with the same number of distribution of spines than protibiae. Metatibiae about 5.3–5.4 times longer than wide (Fig. 12D) and 1.75 times longer than protibiae, with 1 small dorsal spine (which can be absent) and 4 ventral, two of them inserted very apically on the article. Ventral spines shorter than or as long as the diameter of the tibiae. Tibiae about 1.5–1.6 times longer than the first article of the metatarsi. Metatarsi about 1.3–4.0 times longer than tibiae. Praetarsi with 3 simple claws, the median one shorter than the lateral ones.

Urotergites covered by scales; dorsal and ventral scales similar. Dorsal scales make difficult to discern a faint suture between the tergite and the paratergite. Some abdominal tergites are damaged in the holotype and the urotergal chaetotaxy is more visible in the paratype. First urotergite (Fig. 12E) with several small setae inserted in the disc, the remaining urotergites with very few, or completely devoid of, discal setae. The posterior margin of urotergites with 4–5 + 4–5 isolated bifid macrosetae (most of them lost and only their insertions are visible) and with some thin and short acute setae, those of the infralateral region longer (Fig. 13A).

Urotergite X of the male (Fig. 13C) with concave and rounded hind margin and two posterolateral projections which are curved downwards. The posterior part of the tergite bears ventrally 7+7 pegs (on each side, 3 inserted in the posterolateral projection and 4 near the lateral margin of the tergite; on the left side the anterior peg is smaller and thinner than the others, tending to a spiniform shape). Disc of the tergite nearly devoid of setae, only some insertions are visible near the posterior notch and in lateral margins. There are some small and thin setae in these margins, one of them near the apex of the posterolateral projections.

Urotergite X of the female without pegs, its hind margin with a shallow concavity and 1+1 macrosetae inserted in the posterolateral angles (Fig. 13B); the disc, as in the male, nearly without setae.

Urosternite I broken in both available specimens, but the sutures delimiting laterocoxites are visible in the holotype, as well as the setation of the hind margin, consisting in few small setae in the median region and some others in the lateral part (Fig. 13D). Eight pairs of styli, inserted on urosternites II-IX. Eversible vesicles present in urosternites II-VI and pseudovesicles in the urosternite VII. Setation of urosternites II-VII as in Fig. 13E, with 1+1 discal macrosetae, some small setae on the disc, and the hind margin with 1+1 submedian macrosetae (between both vesicles), 1+1 sublateral macrosetae (inserted between vesicles and the basis of the styli) and several acute setae in the outer part, more lateral than styli.

Urosternite VIII of the male entire, of females divided in free coxites. Subgenital plate of the female triangular, with acute hind margin, wider than long (ratio length/width about 0.75), with 1+1 short discal macrosetae (bifid) and several setae on the lateral margins (Fig. 13F).

The genital region of both specimens is damaged, so in the male the hind margin of the urosternite VIII is not visible and the penis and the paramera are lost, and the ovipositor of the female is broken basally, so the length, number of divisions (only 5 are preserved) and the characteristics of the apices of gonapophyses are not known. Terminal filaments probably long but broken basally in the available specimens; maximum preserved length is 0.5 mm. Cerci with 5 acute pegs, longer and thinner than those on the urotergite X (Fig. 14A, B), 1–2 on the proximal division, 3 on the second division and 0–1 on the third division, arranged in a single row. The short basal part of the paracercus that is preserved shows one spiniform small peg in the apical limit of the basal division, the second division is lost (Fig. 14C).

###### Discussion.

This new species of Lepidosporacan be assigned to thesubgenusBrinckina Wygodzinsky, 1955 because the absence of scales on the head, so it is compared with the previously described species of this subgenus. Unfortunately, some characters considered important in the taxonomy of Nicoletiidae cannot be used for this comparison because of the damaged state of the abdominal regions of the two available specimens. In spite of this, the characters described above are enough to state that Lepidospora (Brinckina) momtaziana sp. n. differs from all previously known species of the subgenus Brinckina. We present a comparison with seven well distinguished and taxonomically non-problematic species, since the status of L. (B.) “*meridionalis*” var. Silvestri, 1913 and L. (B.) hemitrichavar.progressa Silvestri, 1942 remains doubtful and requires further studies, as Mendes (2002) discussed.

With the previously mentioned limitations, three characters have been considered significant to distinguishing between the new Iranian species and the remaining taxa of the subgenus: the shape and length/width ratio of the pedicellar apophyses of males, the setation of the disc of the nota and the number and arrangement of pegs in terminal filaments (mainly in cerci). The comparison based on these characters is summarized in Table 3. More details about the comparison with each of the species are given below.

It worth mentioning that the shape and length/width ratio of the pedicellar apophysis, a distinctive character that can be used only in adult male specimens, should be described carefully. As Molero et al. (2013) stated, “similar antennae placed in different positions could be interpreted as different shapes, but upon rotating the antennae, similar structures can be recognized”. Antennae that are presumably considered as symmetrical show different appearances when illustrated in different positions; for example, see the drawings of Smith and McRae (2016) of the left and right pedicellar apophyses of two male specimens of the same species: are they different because an intraspecific variability or because of being drawn in different positions? If we consider the form that can be interpreted from the drawings of the left apophysis, the length/width ratio is about 2.3, but this parameter is approximately 3 in the drawing of the right apophysis. They are similar in length at the same scale; our experience suggests that both apophyses are identical, but the shape of the transversal section of the apophysis is not circular but elliptical; if the left apophysis seems to be wider it is because it is drawn in a different position (seen from the wider diameter of the ellipse) than the right (seen from the narrower diameter). In the new species from Iran, differences between both pedicellar apophyses are not completely explained by different positions (in the slide), so we conclude that they are actually asymmetrical; the difference is not based only in the length/width ratio but also on the shape of the apical part (narrower than the basal in the left apophysis) and on the different chaetotaxy (being aware of the fact that insertions of detached setae must be accounted for).

The following comparisons of other previously described species are based on illustrations from their original descriptions.

Lepidospora (B.) makapaan Wygodzinsky, 1955 from South Africa (Transvaal) has a lot of pegs on the paracercus but lacks pegs on cerci, a character not shared with any other species of the subgenus, including the new species. Moreover, the chaetotaxy of the head and urosternite I is denser in the South African species and the subgenital plate of females is more rounded apically. Adult specimens of L. (B.) makapaan are clearly bigger than adults of the Iranian species, since females of the South African species can reach 16 mm length and their tibia are more than 7 times longer than wide (less than 6 in the new species).

Lepidospora (B.) relicta Smith & McRae, 2016 from northwestern Australia is probably the representative of the subgenus that shows more affinities with the new species from Iran. It is similar because it has pegs on cerci and paracercus and sparse setulae on discs of nota, but males show symmetrical and longer pedicellar apophyses. The number of pegs of the urotergite X is higher (20 against 14 in the new species) and the paracercus (=appendix dorsalis) has 2+2 pegs in the two basal divisions (the Iranian species has only one spiniform seta that can be considered as a peg since it is modified in respect to the remaining setae of the appendix, but nothing can be said about the second division because it is lost).

Males of L. (B.) hamata Mendes, 2002 from Congo has a shorter pedicellar apophysis, which is near to the length/width ratio of L. (B.) momtaziana sp. n., but the shape is different and apophyses seem to be symmetrical; the glandular seta is almost apical in the African species and more subapical in L. (B.) momtaziana sp. n. Moreover, the urotergite X of the male is quite distinct and the species from Congo lacks pegs in the paracercus and the new species shows, at least, one spiniform peg.

Lepidospora (B.) garambensis Mendes, 2002, described from Congo too, is also devoid of pegs in the paracercus and the apophysis of males is longer. Moreover, mesotibiae of L. (B.) garambensis have short strong ventral spines which are absent in the new species.

Males of L. (B.) alticola Wygodzinsky, 1965 (from Kenya) are not known. Comparing females of this species with the new one, the labial palp of this African species is stouter, as long as wide (at least 1.5 times longer than wide in L. (B.) momtaziana sp. n.) and the discal setae of nota are more abundant and longer (about 1/8 of the total length of the nota in the African species and 1/20 in the Iranian species).

Lepidospora (B.) hemitricha Silvestri, 1942 and L. (B.) hemitrichoides Wygodzinsky, 1962 (from China and Afghanistan, respectively) are similar in bearing pegs on cerci and paracercus, and their apophysis have a similar length/width ratio (approximately 2), but the setation of the disc of their nota is stronger (high number of setae and most setae are longer). Additionally, their urotergites X have a higher number of pegs (12+12 in *L.hemitricha* and about 17+17 in *L.hemitrichoides*) and the pegs on cerci are more spiniform (long and acute) in shape. In *L.hemitricha*, the length/width ratio of their tibiae is lower (about 4 in metatibiae) than in the new species from Iran and males of *L.hemitrichoides* have longer (finger-like) posterolateral angles of the urotergite X, surrounding a deeper median notch, and the number and distribution of spiniform pegs in the basal division of the paracercus is clearly different from the new species (a lot of pegs in three rows in the Afghan species).

Considering the limitations derived from the lack of information about males of *L.alticola* and the doubtful status of some species (see above), *Lepidosporamomtaziana* sp. n. can enter in the key of *Lepidospora* made by Mendes (2002) in at step 34 with the following modifications:

**Table:** 

34	Nota (mainly pronotum) with numerous setae on disc. Ovipositor long, surpassing stylets IX by about 4 times their length. Pegs along male cerci and paracercus. Asiatic species	**35**
34’	Nota with few scattered setae on disc (they can be a little more abundant in the anterior part of the pronotum). Ovipositor similar or shorter (its length in L. (B.) montaziana not known)	**34A**
34A	Setae on the disc of nota very short, about 1/20 of the length of the notum. Iranian species	***Lepidosporamomtaziana* sp. n.**
34A’	Setae on the disc of nota longer. African species	**36**

###### Distribution.

Known only from the type locality, Momtaz Cave, in Fars province, Iran.

###### Etymology.

The specific name refers to the cave where specimens have been collected. Momtaziana refers to Momtaz, in genitive case, with a feminine ending (-iana) that means “belonging to”.

###### Habitat.

This new species has been collected in the hypogean zone (complete darkness) of Momtaz Cave.

## Conclusions

As a result of the surveys performed in caves in Iran, several new taxa of basal Hexapoda (Microcoryphia and Zygentoma) have been described or reported for the first time in this country. Some of them can be considered strictly subterranean, but others are facultative in this environment (they are usually epigean or epiedaphic insects but in the climatic conditions of southern Iran they avoid surficial environments and hide at deeper levels of soil or in cavities).

Previous to our studies on subterranean basal hexapods, only five species of Microcoryphia were known from Iran (Table 4), all of them belonging to the family Machilidae; four of them are endemic to the Middle-East region (Lepismachilis (L.) hobertandti Wygodzinsky, 1952; *Silvestrichiliswittmeri* Bitsch, 1970; *Machilanusspinosissimus* Mendes, 1981 and L. (L.) dominiaki Mendes, 1985) and the fifth, *Trigoniophthalmusalternatus* (Silvestri, 1904), with a wide distribution over continental Europe, reaching Spain to the west. *Haslundiellairanica* sp. n. represents the sixth species of Microcoryphia known for Iran.

Considering Zygentoma, 14 species of this order were known from Iran in surficial environments, but adding the species described by us in this work and those reported before in a previous paper (Tahami et al. 2018), the number reaches 20, which represents an increase of about 40% in the knowledge of this order (see Table 4).

Moreover, we think that further surveys in other regions and caves of Iran could increase significantly this diversity.

Finally, a key for the eight subterranean basal insects known to date from Iran is given below. For Zygentoma, this key can be considered as complementary to that provided by Kahrarian et al. (2014) for epigean Lepismatidae.

**Table:** 

1	Body not flattened, with humped thorax. Paracercus considerably longer than the two cerci. Head with big compound eyes and also with ocelli (order Microcoryphia, family Machilidae)	***Haslundiellairanica* sp. n.**
–	Body more or less dorsoventrally flattened, the thorax is not humped. Paracercus subequal in length or longer than cerci. Head usually without ocelli and sometimes without eyes (order Zygentoma)	**2**
2	Compound eyes present, small, with about 13 ommatidia. Male pedicellus without apophysis. Urosternite VIII of females divided in two coxites. Hind margins of urosternites with macrosetae arranged in dense groups forming one row (comb) and without vesicles (family Lepismatidae)	**3**
–	Eyes absent. Male pedicellus with an apophysis. Urosternite VIII of females divided in two coxites and a submedian subgenital plate. Hind margins of urosternites with isolated macrosetae, not forming combs, some of them with a pair of sublateral vesicles (family Nicoletiidae)	**6**
3	Prosternum strongly reduced. Ovipositor apically provided with strong sclerotized teeth. subfamily Acrotelsatinae. Urotergite X acutely triangular.	***Acrotelsacollaris* (Fabricius, 1793)**
–	Prosternum normally developed. Ovipositor usually without spines (primary type). Urotergite trapezoidal, with their hind margin straight or slightly convex or concave, but not acute	**4**
4	With smooth, apically bifid macrosetae. Males with paramera (subfamily Lepismatinae). Hind margin of urotergites I-IX with isolated macrosetae, at most with an infralateral group of 2–3 macrosetae. Urotergite X longer than wide and with concave hind margin	***Neoasterolepismapalmonii* (Wygodzinsky, 1942)**
–	With feathered macrosetae. Males without paramera. Urotergites I-VIII and X with at least 1+1 combs of macrosetae. Urotergites II-V with 3+3 combs of macrosetae. Urotergite IX without setae. Urotergite X wider than long, with straight hind margin or slightly concave (subfamily Ctenolepismatinae, genus *Ctenolepisma*)	**5**
5	Urosternites without median combs of macrosetae. Urotergite VI with 3+3 combs of macrosetae	***Ctenolepismasubterraneum* sp. n.**
–	Urosternites II-VI with one median comb of macrosetae. Urotergite VI with 2+2 combs of macrosetae	***Ctenolepismatargionii* (Grassi & Rovelli, 1889)**
6	Body short, with short antennae and terminal filaments (shorter than half the body length). Pronotum clearly wider than head, abdomen width tapering backwards. Urosternite I entire … subfamily Atelurinae. All the body covered (including head) with scales	***Persiatelurinafarsiana* Molero, Tahami, Gaju & Sadeghi, 2018**
–	Body subcylindrical, long, with antennae and terminal filaments, when well preserved, longer than body or slightly shorter. Pronotum as wide as or slightly wider than head, abdomen width not clearly tapering backwards. Urosternite I divided in a median sternite and two laterocoxites … subfamily Coletiniinae, genus *Lepidospora*. Head without scales	**7**
7	Thorax with scales	***Lepidospora*** (***Brinckina***) ***momtaziana* sp. n.**
–	Thorax without scales	***Lepidospora*** (***Brinckiletinia***) ***malousjanica* Molero et al., 2018**

**Table 4.** Faunistic progress in Iran, comparing the number of species of Microcoryphia and Zygentoma previously known in this country before and after the present survey of subterranean habitats.

Family	Number of species previously known in Iran	Number of species from in Iran after the present survey	Increase of diversity
Machilidae (order Microcoryphia)	5	6	1 (20%)
Lepismatidae (order Zygentoma)	13	16	3 (23%)
Nicoletiidae (order Zygentoma)	0	3	3 (new family for Iran)
Protrinemuridae (order Zygentoma)	1	0	0
Total	19	26	7 (37%)

## Supplementary Material

XML Treatment for Haslundiella iranica

XML Treatment for Ctenolepisma subterraneum

XML Treatment for Acrotelsa collaris

XML Treatment for Neoasterolepisma palmonii

XML Treatment for Ctenolepisma (Sceletolepisma) targionii

XML Treatment for Lepidospora (Brinckina) momtaziana
